# Lack of association of the cyclin D1 G870A variation with oral carcinoma risk: Evidence from 2,404 subjects

**DOI:** 10.3892/etm.2012.648

**Published:** 2012-07-24

**Authors:** XIAN-LU ZHUO, JUN-JUN LING, HOU-YU ZHAO, YAN ZHOU, YU-FENG SONG, YING-HUI TAN

**Affiliations:** 1 Department of Stomatology, Xinqiao Hospital, Third Military Medical University, Chongqing;; 2 Affiliated Hospital of Guiyang Medical College, Guiyang;; 3 Department of Otolaryngology, Southwest Hospital, Third Military Medical University, Chongqing, P.R. China

**Keywords:** cyclin D1 G870A, mouth neoplasm, susceptibility, meta-analysis, polymorphism

## Abstract

Evidence implicates cyclin D1 (CCND1) G870A polymorphisms as risk factors for various cancers. An increasing number of investigations have been conducted on the association of CCND1 G870A polymorphisms with susceptibility to oral carcinoma, and have yielded inconclusive results. The aim of the present study was to derive a more precise estimation of the correlation. Meta-analyses examining the association between CCND1 G870A polymorphisms and oral carcinoma were performed. Separate analyses on ethnicity, smoking status and control sources were also implemented. Eligible studies were identified prior to February 2012. From the overall data from 1,128 cases and 1,276 controls, no associations of CCND1 G870A polymorphisms with oral carcinoma were observed [AA vs. GG: odds ratio (OR)=1.06; 95% confidence interval (CI), 0.62–1.82; dominant model: OR=1.04; 95% CI, 0.76–1.43; recessive model: OR=1.06; 95% CI, 0.70–1.59]. In the subgroup analysis by ethnicity, smoking status and control sources, no significant associations of CCND1 G870A polymorphisms and oral cancer were observed for the three genetic models. Collectively, the data failed to suggest CCND1 G870A polymorphism as a low-penetrant risk factor for developing oral carcinoma. Additional studies with large sample sizes concerning different ethnicities in different areas are required.

## Introduction

Oral squamous cell carcinoma is one of the most common cancers in the world and its main risk factors are cigarette smoking and alcohol consumption (1,2) as well as betel quid chewing (3). Increasing epidemiological evidence suggests their complex interactions between numerous genetic and environmental factors are major causes. Although individuals are exposed to environmental risk factors and extensive tobacco, alcohol consumption and betel quid chewing, oral cancer develops only in a small proportion of exposed individuals, implying that genetic factors may play a role in its carcinogenic mechanisms.

Our previous meta-analyses revealed GSTM1 genetic variation as a risk factor for oral cancer, whereas the CYP1A1 (4) or TP53 (5) polymorphism may not increase the susceptibility to oral neoplasm. In addition, meta-analyses conducted in 2009 and 2011, respectively, suggested XRCC1 (6) and CYP2E1 (7) polymorphisms as biomarkers for oral carcinoma in Asian populations. However, although genetic factors are important, only a few genes associated with genetic susceptibility to oral cancer were identified.

Cyclin D1 (CCND1) is a key regulatory protein that plays an important role in the transition from G1 to S phase of the cell cycle during cell division. Inhibition of CCND1 function often results in cell cycle arrest, whereas overexpression of CCND1 may disrupt normal cell cycle control and subsequently contribute to oncogenesis (8). Evidence suggests that gene amplification and protein overexpression of CCND1 were detected in a number of cancers, including oral cancer (9–11), and were considered to be associated with a poor prognosis. Hence, CCND1 has been regarded as a potential target for tumor therapy (12,13).

Previously, a common functional polymorphism, G870A, of CCND1 has been widely studied as a possible low-penetrant susceptibility allele for various cancers (14). CCND1 G870A is a silent variant that does not result in an amino acid alteration within the protein sequence. The A allele has been considered to result in an alternatively spliced transcript of CCND1, namely, transcript b. This alternate transcript lacks exon 5 of CCND1 that contains a PEST-rich region, critical for the degradation of CCND1. Thus, transcript b (A allele) has been shown to have a longer half-life than the transcript a (G allele, the wild-type gene) encoded protein, resulting in different biochemical and biological protein features (15). It has been demonstrated that transcript b is more likely to bypass the G1-S cell cycle checkpoint, which consequently leads to carcinogenesis (16). As a result, three distinct genotypes were created, namely, homozygous AA, homozygous GG and heterozygous AG. Evidence suggests that the CCND1 polymorphism may have a correlation with its overexpression in cancers (17,18). Thus, evaluation of the association of CCND1 polymorphism with oral cancer risk is required.

Published data on the possible association of CCND1 G870A polymorphism with oral carcinoma have generated inconclusive results. To the best of our knowledge, the question of whether the CCND1 G870A polymorphism increases oral cancer risk remains uncertain. To clarify this association may elucidate the possible risk of oral cancer and therefore contribute to its prevention. As a single study may have been underpowered in clarifying the associations of CCND1 G870A polymorphisms with oral carcinoma susceptibility, in the present study we performed evidence-based quantitative meta-analyses that increased statistical power to address this controversy.

## Materials and methods

### Literature search strategy

We carried out a search in the Medline, EMBASE, OVID, Sciencedirect and CNKI databases without a language limitation, including all manuscripts published until February 2012, with a combination of the following keywords: cyclin D1, CCND1, oral, mouth neoplasm, tumor, cancer, variation and polymorphism. All searched studies were retrieved and the bibliographies were checked for other relevant publications. Review articles and bibliographies of other relevant studies identified were hand-searched to find additional eligible studies.

### Inclusion criteria

The following criteria were used for the literature selection: First, studies should concern the association of CCND1 G870A polymorphism with oral cancer risk; second, studies should be observational studies (case-control or cohort); third, manuscripts must offer the size of the sample, odds ratios (ORs) and their 95% confidence intervals (CIs), the genetic distribution or the information to infer the results. After searching, we reviewed all manuscripts in accordance with the criteria defined above for further analysis.

### Data extraction

Data were extracted from all eligible publications independently by two of the authors according to the inclusion criteria mentioned above. For conflicting evaluations, an agreement was reached following a discussion. If a consensus could not be reached, another author was consulted to resolve the dispute and then a final decision was made by the majority of the votes. The extracted information was entered into a database. For data not provided in the main text, the relevant information was obtained by contacting corresponding authors if and when possible.

### Statistical analysis

The OR of CCND1 G870A polymorphisms and oral cancer was estimated for each study. The pooled ORs were calculated for the additive model (AA vs. GG), dominant model (AA+AG vs. GG) and recessive model (AA vs. AG+GG), respectively. For detection of any possible sample size biases, the OR and its 95% CI from each study was plotted against the number of participants. A Chi-square based Q-statistic test was performed to assess heterogeneity. If the result of the heterogeneity test was P>0.1, ORs were pooled according to the fixed-effects model (Mantel-Haenszel), otherwise, the random-effects model (DerSimonian-Laird) was used. The significance of the pooled ORs was determined by Z-test. The Hardy-Weinberg equilibrium (HWE) was assessed via Fisher’s exact test.

Publication bias was assessed by visual inspection of funnel plots (19), in which the standard error of log (OR) of each study was plotted against its log (OR). An asymmetric plot indicates a possible publication bias. The symmetry of the funnel plot was further evaluated by Egger’s linear regression test (20). Statistical analysis was undertaken using the program Review Manager 5 and Stata 11.0 softwares (Stata Corporation, College Station, TX, USA).

## Results

### Study characteristics

Publications relevant to the key words were retrieved and screened. A total of 52 studies on CCND1 were searched and screened for retrieval, of which 43 irrelevant studies were excluded. Two studies (21,22) were then excluded since the data were not confined to oral cancer. One study (23) was excluded due to it being a review and six studies (17,24–28) were excluded since they concerned only CCND1 expression but not its polymorphism. A total of six case-control studies (29–34) were selected.

All the included six studies were written in English. We established a database according to the extracted information from each article. The relevant information is listed in Table I. The first author and the number and characteristics of cases and controls for each study as well as other necessary information are presented.

Of the included studies, two studies were on Caucasian (32,34), three on Asian (29,31,33) and one on mixed ethnicity populations (30). Data with regard to smoking status was available in two studies (29,32).

As shown in Table II, the distributions of the CCND1 G870A genotype of the included studies are also presented. The genetic distributions of the control groups of two studies (29,30) deviated from HWE, while the remaining studies did not.

### Test of heterogeneity

We analyzed the heterogeneity of an additive model (AA vs. GG), dominant model (AA+AG vs. GG) and recessive model (AA vs. AG+GG).

As shown in Table III, the heterogeneity for the overall data with regard to oral cancer was significant in additive, dominant and recessive models since the P-values were <0.1 for Q-tests. However, subgroup analyses with regard to ethnicity, smoking status and source of control were conducted. The P-value for heterogeneity indicated a reduced or removed heterogeneity when the data were divided into relevant subgroups.

### Meta-analysis results

**Figure 1:**
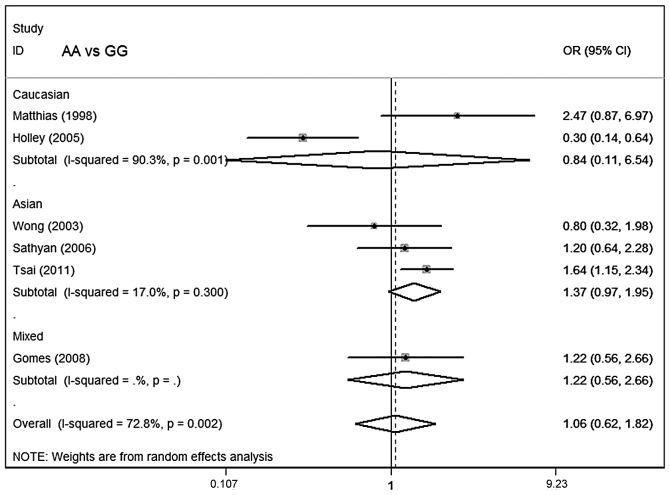
Meta-analysis for the association of oral cancer risk with CCND1 polymorphisms (AA vs. GG).

**Figure 2:**
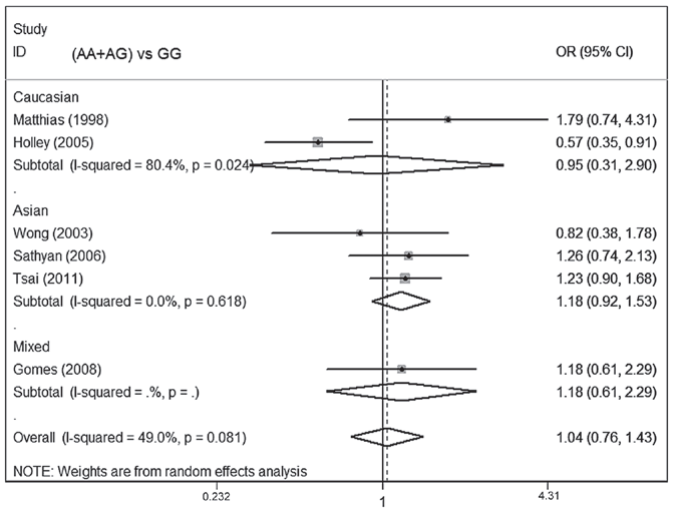
Meta-analysis for the association of oral cancer risk with CCND1 polymorphisms (AA+AG vs. GG).

**Figure 3:**
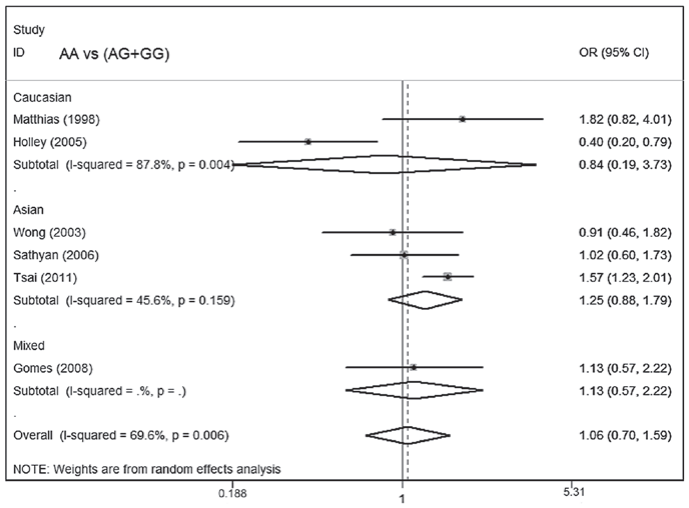
Meta-analysis for the association of oral cancer risk with CCND1 polymorphisms (AA vs. AG+GG).

Table III lists the main results of the meta-analysis for oral cancer. The overall data did not demonstrate any association of CCND1 polymorphism with oral cancer risk (AA vs. GG: OR=1.06; 95% CI, 0.62–1.82; P=0.002 for heterogeneity; dominant model: OR=1.04; 95% CI, 0.76–1.43; P=0.08 for heterogeneity; recessive model: OR=1.06; 95% CI, 0.70–1.59; P=0.006 for heterogeneity; Figs. 1–3).

In subgroup analysis stratified by ethnicity, no significant associations were identified among Asians, Caucasians or mixed ethnicities for the three genetic models. In subgroup analysis with regard to smoking status and control source, no significant association of the CCND1 polymorphism with oral cancer susceptibility was observed.

### Sensitivity analysis

When the effects-models were changed, the significance of the overall data for the three models was not statistically altered (data not shown). We then excluded two studies (29,30) whose genetic distributions in controls exhibited a marked deviation from HWE, given that the deviation may contribute to any bias (35). The significance of the overall data in the three models was also not statistically altered, confirming the stability of the results. Hence, results of the sensitivity analysis suggest that the data in this meta-analysis are relatively stable and credible.

### Bias diagnostics

**Figure 4:**
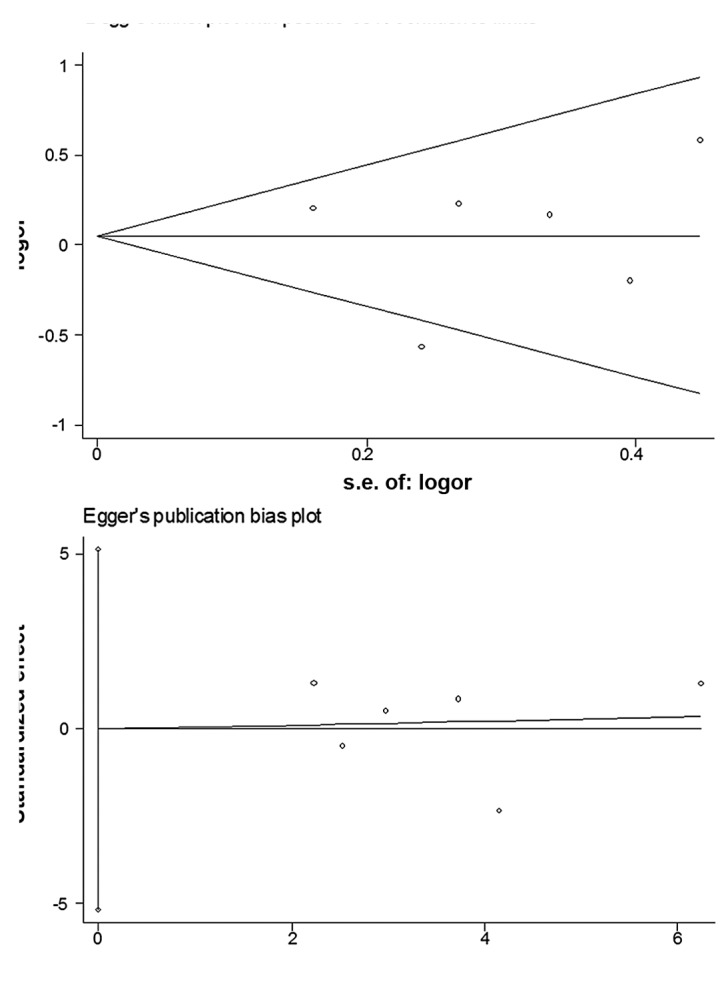
Funnel plot and Egger’s linear regression test (AA+AG vs. GG).

Funnel plots were created for the assessment of possible publication biases. Egger’s linear regression tests were used to assess the symmetry of the plots (Fig. 4). The data suggest that the funnel plots were symmetrical for the three models (AA vs. GG: t=−0.88, P>0.05; dominant model: t=−0.01, P>0.05; recessive model: t=−1.55, P>0.05), suggesting that the results of these meta-analyses are relatively stable and the publication biases do not have an evident influence on the results of the meta-analyses.

## Discussion

In the present study, the results of meta-analyses failed to reveal a significant association of CCND1 G870A polymorphism with oral cancer risk.

Recently, several meta-analyses have been conducted to assess the association of CCND1 G870A polymorphisms with cancer risks. The A allele has been suggested to increase risk of digestive tract (36) and colorectal cancer (37) in Caucasian populations. Similarly, the A allele appears to increase cancer risk in Chinese populations for breast (38) and bladder cancer (39), indicating that A allele carriers may produce an elevated cancer risk. However, a meta-analysis conducted on cervical cancer (40) failed to show this marked association. Similarly, no evidence supports the association of CCND1 genetic variation with head and neck cancer (41) in a recently published meta-analysis, of which the data were combined as head and neck cancer rather than oral carcinoma, and two important studies (29,32) on oral cancer that met the inclusion criteria were ignored. In the present study, we focused on oral cancer and screened the literature to include possible studies. In the present study, in addition to ethnicity, more subgroups with regard to smoking status and source of controls were further assessed. No marked increased oral cancer risk was observed in any comparisons for the three genetic models.

Smoking is a well-known risk factor for a number of cancers, including lung and oral cancer. Effects of interactions between smoking and the CCND1 genotype on lung cancer risk have been suggested previously (42,43). In this study, no increased cancer risk was demonstrated in the smoking subgroup analysis (Table III), suggesting that smoking did not modify the effect of CCND1 polymorphisms on oral cancer risk. Notably, only two studies (29,32) containing 790 cases and 930 controls were available for the subgroup analysis. Further studies with large sample sizes assessing smoking status are required for further clarification.

Between-study heterogeneity was observed in Table III and thus random-effects models were used. We further deselected the studies (29,30) whose controls deviated from the HWE and observed that the heterogeneity was not removed. Additionally, the significance of the overall data was not statistically altered. Therefore, these two studies were not excluded from the present study. However, the P-value for heterogeneity indicated a reduced or removed heterogeneity when the data were divided for subgroup analysis.

Publication biases were assessed via funnel plots and their symmetries were further evaluated by Egger’s linear regression tests. No evident biases were observed, suggesting little influences of publication biases on the results.

Several limitations may be included in the present study. First, in this meta-analysis, the majority of published studies were written in English, Chinese and Portuguese, as cited by the databases consulted. It is possible that certain published or unpublished studies written in other languages that met the inclusion criteria were missed. Hence, inevitable publication biases may exist, although the funnel plots and Egger’s linear regression tests implied no marked publication biases. Second, the subgroup analysis concerned only Caucasians, Asians and mixed ethnicities. Data with regard to other ethnicities were not identified. The Asian populations were confined to Indians and Chinese in Taiwan. Studies conducted in Southeast Asia, Japan and mainland China are required to increase statistical power for the demonstration of this association. Third, hospital-based controls were used in some included studies. Hence, non-differential misclassification bias may exist. However, subgroup analysis was carried out and no evident influence on the results was identified. Gene-gene and gene-environment interactions should also be considered in further studies. However, the sensitivity analysis and publication bias analysis showed the stability and credibility of the present meta-analysis.

In summary, despite the limitations, the results of the present meta-analysis failed to suggest a significant association between CCND1 G870A genetic variations and oral cancer risk.

## Figures and Tables

**Table I:** Characteristics of the studies included in the meta-analysis.

First author	Year of publication	No. of cases (male/female)	No. of controls (male/female)	Type of controls	Age range (mean), years		Country
					Cases	Controls	
Matthias (34)	1998	38 (25/13)	191 (149/42)	191 non-cancerous controls (hospital-based)	NA (59.29)	NA (NA)	Germany
Wong (33)	2003	70 (65/5)	93 (74/19)	93 controls (population-based)	31–82 (53.3)	22–75 (49.4)	China
Holley (32)	2005	174 (135/39)	155 (125/30)	155 non-cancerous controls (hospital-based)	NA (55.6)	NA (59.9)	Germany
Sathyan (31)	2006	176 (119/57)	142 (98/44)	142 non-cancerous controls (age-, gender-matched; hospital-based)	36–85 (59)	35–80 (58)	India
Gomes (30)	2008	80 (60/20)	80 (59/21)	80 healthy individuals (age-, gender, tobacco usage-matched; population-based)	39–82 (57.5)	39–85 (53.5)	Brazil
Tsai (29)	2011	620 (586/34)	620 (586/34)	620 non-cancerous controls (age-, gender-, habit-matched; hospital-based)	NA (52.4)	NA (51.3)	China

NA, not available.

**Table II:** Distribution of the CCND1 genotype among oral cancer cases and controls included in the meta-analysis.

First author	Genotyping method	Cases			Controls			HWE (control)	
		AA	AG	GG	AA	AG	GG	Chi-square	P-value
Matthias	PCR-RFLP	11	20	7	35	101	55	0.918	>0.05
Wong	PCR-SSCP	19	36	15	27	49	17	0.406	>0.05
Holley	PCR-RFLP	14	94	66	28	87	40	2.593	>0.05
Sathyan	PCR-SSCP	39	71	36	36	61	40	1.620	>0.05
Gomes	PCR-RFLP	25	30	25	23	29	28	5.926	<0.05
Tsai	PCR-RFLP	213	323	84	155	365	100	21.625	<0.05

HWE, Hardy-Weinberg equilibrium.

**Table III:** Main results of the pooled data in the meta-analysis.

Variable	No. (cases/controls)	AA vs. GG			(AA+AG) vs. GG			AA vs. (AG+GG)		
		OR (95% CI)	P-value	P-value (Q-test)	OR (95% CI)	P-value	P-value (Q-test)	OR (95% CI)	P-value	P-value (Q-test)
Total	1128/1276	1.06 (0.62–1.82)	0.83	0.002	1.04 (0.76–1.43)	0.80	0.08	1.06 (0.70–1.59)	0.80	0.006
Ethnicity										
Caucasian	212/346	0.84 (0.11–6.54)	0.87	0.001	0.95 (0.31–2.90)	0.93	0.02	0.84 (0.19–3.73)	0.82	0.004
Asian	836/850	1.37 (0.97–1.95)	0.08	0.3	1.18 (0.92–1.53)	0.20	0.62	1.25 (0.88–1.79)	0.22	0.16
Mixed	80/80	1.22 (0.56–2.66)	0.62	NA	1.18 (0.61–2.29)	0.61	NA	1.13 (0.57–2.22)	0.73	NA
Smoking status										
Ever smoking	602/588	0.55 (0.04–7.12)	0.65	0.000	0.83 (0.32–2.20)	0.71	0.002	0.62 (0.07–5.38)	0.66	0.000
Never smoking	188/342	1.16 (0.65–2.08)	0.61	0.71	1.01 (0.61–1.67)	0.96	0.75	1.22 (0.81–1.85)	0.34	0.82
Source of control										
Hospital-based	978/1103	1.09 (0.50–2.38)	0.83	0.001	1.06 (0.68–1.66)	0.78	0.03	1.06 (0.59–1.91)	0.85	0.002
Population-based	150/173	1.02 (0.56–1.84)	0.96	0.49	1.02 (0.61–1.68)	0.95	0.48	1.02 (0.63–1.65)	0.95	0.67

NA, not applicable.
